# Genetic Variations Associated with Vitamin A Status and Vitamin A Bioavailability

**DOI:** 10.3390/nu9030246

**Published:** 2017-03-08

**Authors:** Patrick Borel, Charles Desmarchelier

**Affiliations:** NORT, Aix-Marseille Université, INRA, INSERM, 13005 Marseille, France; patrick.borel@univ-amu.fr (P.B.); charles.desmarchelier@univ-amu.fr (C.D.); Tel.: +33-4-9132-4277 (P.B.)

**Keywords:** genetic polymorphisms, absorption, bioavailability, β-carotene, retinyl palmitate, retinol, nutrigenetics, blood concentration, provitamin A, carotenoids, β-cryptoxanthin, α-carotene, postprandial

## Abstract

Blood concentration of vitamin A (VA), which is present as different molecules, i.e., mainly retinol and provitamin A carotenoids, plus retinyl esters in the postprandial period after a VA-containing meal, is affected by numerous factors: dietary VA intake, VA absorption efficiency, efficiency of provitamin A carotenoid conversion to VA, VA tissue uptake, etc. Most of these factors are in turn modulated by genetic variations in genes encoding proteins involved in VA metabolism. Genome-wide association studies (GWAS) and candidate gene association studies have identified single nucleotide polymorphisms (SNPs) associated with blood concentrations of retinol and β-carotene, as well as with β-carotene bioavailability. These genetic variations likely explain, at least in part, interindividual variability in VA status and in VA bioavailability. However, much work remains to be done to identify all of the SNPs involved in VA status and bioavailability and to assess the possible involvement of other kinds of genetic variations, e.g., copy number variants and insertions/deletions, in these phenotypes. Yet, the potential usefulness of this area of research is exciting regarding the proposition of more personalized dietary recommendations in VA, particularly in populations at risk of VA deficiency.

## 1. Introduction

The term vitamin A (VA) is employed generically for all derivatives of β-ionone (other than the carotenoids) that possess the biological activity of all-*trans* retinol (RET) or are closely related to it structurally [1]. This encompasses a group of fat-soluble molecules that are found as preformed VA (mainly RET and its esters, retinal and retinoic acid) in animals and animal products and as provitamin A (proVA) carotenoids in fruit and vegetables. VA is essential to human health and is involved in many metabolic and physiological processes, such as vision [2,3,4,5], cell differentiation [6,7], embryonic development [8,9] and immunity [10]. VA deficiency is still a serious public health problem in developing countries, where it still affects about one-third of children [11]. It greatly increases the severity of common childhood infections (e.g., measles, malaria) by compromising the immune system. Symptoms include impaired vision, in extreme cases irreversible blindness, impaired epithelial integrity, exposing the affected individuals to infections and reduced immune response. Night blindness is estimated to affect 250,000–500,000 children each year, of which 50% die within the following year. VA deficiency also contributes to the global burden of growth retardation, which affects 160 million children under five.

The current recommended dietary allowance in France is 600 µg RET activity equivalents (RAE) per day for women and 800 μg RAE/day for men. International committees have established RAE, considering the variability in carotenoid bioavailability depending on the matrix in which they are incorporated, as follows:

- 1 µg RAE = 1 μg RET
- 1 µg RAE = 2 µg all-*trans* β-carotene (βC) from supplements
- 1 µg RAE = 12 µg of all-*trans* βC from food
- 1 µg RAE = 24 µg α-carotene or β-cryptoxanthin from food
- 1 µg RAE = 3.33 IU RET

Although it is well established that there is an insufficient VA intake in developing countries, usually due to an insufficient availability of VA-rich foods (i.e., animal products), leading to VA deficiency, recent data have pointed to intakes below recommendation levels in several developed countries (concerning more than 75% of the population aged 19–50 in the U.S.) [12]. In the human diet, most preformed VA occurs as retinyl palmitate (RP), while β-carotene (βC) is the most abundant proVA carotenoid [13]. The proportion of preformed VA and proVA carotenoids that we eat depends on our dietary habits. For example, in vegans, 100% of dietary VA originates from proVA carotenoids. A recent analysis of the results of 11 studies in eight developed countries (representing ≈ 120,000 participants) has shown that preformed VA intake accounted for about 65% of total VA intake, while provitamin A carotenoids represented 35% of total VA intake (βC: 86%; α-carotene: 10%; β-cryptoxanthin: 4% thereof, respectively) [13]. Although both preformed VA and proVA carotenoids can be metabolized to the three main active VA molecules recovered in the human body, i.e., RET, retinal and retinoic acid, the metabolic pathways by which each form of VA is metabolized are partly different until they are converted to retinal. This allows us to suggest that individuals, or populations, that possess different abilities to absorb or metabolize these two forms of VA, due to, e.g., genetic variations that modulate expression/activity of proteins involved in these pathways, are not able to similarly use these two forms of VA. 

This review starts with a description of the fate of VA in the human body, from the food matrix in which it is ingested to extra-hepatic tissues, by going through its main storage organ: the liver. This allows us to identify candidate proteins, and thus candidate genes, that could explain the interindividual variability in blood and tissue concentrations of VA molecules. This review then lists the genetic variations that have been associated with the interindividual variability in VA blood concentration and bioavailability. The review finishes by listing the points to focus on in the forthcoming years to identify the main genetic variations that are involved in these phenotypes.

## 2. Metabolism of Vitamin A in the Gastrointestinal Lumen 

**Figure 1:**
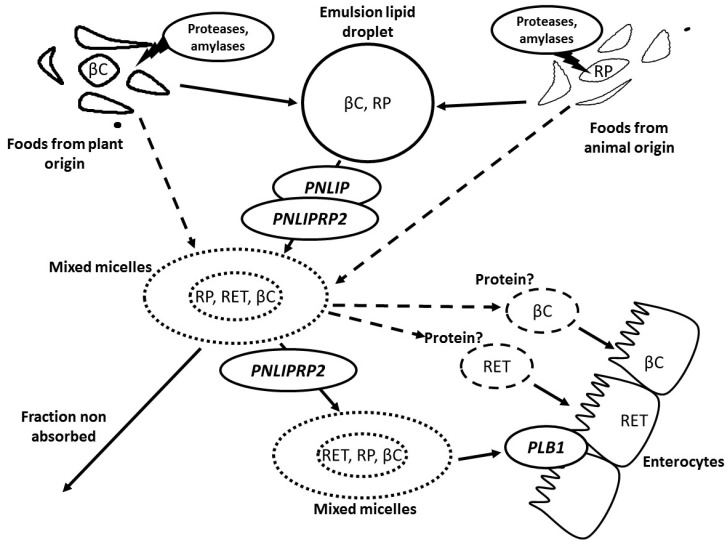
Proteins involved, or hypothesized to be involved, in vitamin A (VA) metabolism within the lumen of the upper gastrointestinal tract. βC: β-carotene and all other provitamin A carotenoids; PLB1: phospholipase B; PNLIP: pancreatic lipase; PNLIPRP2: pancreatic lipase-related protein 2; RET: retinol; RP: retinyl palmitate and all other retinyl esters. Proteins followed by a question mark have been hypothesized to be involved because RET and βC are not soluble in water, and thus, non-micellarized VA is assumed to be associated with proteins. A dotted arrow means the pathway is suspected to exist, but there is no evidence thereof yet.

Dietary preformed VA, which is chiefly RP, and dietary proVA carotenoids, which are chiefly βC, are both insoluble in water. Thus, although they can be ingested in very different food matrices, e.g., butter, liver or carrots, they are assumed to transfer, at least in part, from their food matrix to lipid droplets of dietary fat emulsions that are present in the gastrointestinal lumen during digestion [14,15,16,17] (Figure 1). This transfer, as well as the transfer of VA to mixed micelles, is modulated by numerous factors, e.g., food matrix, food processing, presence of fibers, lipids, etc. It is beyond the scope of this review to describe the current knowledge on all of these factors, but dedicated reviews can be found elsewhere [14,18]. This transfer can be facilitated by gastric and pancreatic enzymes that participate in food digestion, i.e., proteases, amylases and lipases. VA or proVA carotenoids transferred to lipid droplets are then assumed to transfer to mixed micelles, although a fraction might be solubilized by dietary proteins [19]. Again, this transfer is assumed to be facilitated by the action of digestive enzymes [20]. From this step on, RP and βC fates branch off. RP in mixed micelles and in emulsion lipid droplets is hydrolysed to RET by pancreatic lipase (encoded by *PNLIP*) and also to a lesser extent by pancreatic lipase-related protein-2 (encoded by *PNLIPRP2*) [15]. Moreover, the brush border membrane-associated enzyme phospholipase B, encoded by *PLB1*, has also been suggested to participate in RP hydrolysis [21]. It has been shown that inhibition of retinyl ester hydrolysis in the gut dramatically impairs RET absorption [22], adding evidence that retinyl ester hydrolysis is compulsory prior to RET absorption. βC is not significantly metabolized, or chemically modified, and stays as such in mixed micelles [16]. Then, mixed micelles transport RET and βC to the apical side of the enterocyte where they are taken up via both passive diffusion and facilitated transport (see the next section for a state of the art description of these mechanisms). Surprisingly, while two apical membrane proteins involved in the uptake of βC have been identified, the protein(s) involved in the uptake of RET has (have) not. Yet, it has been assumed for forty years that RET uptake is, at least partly, facilitated [23]. 

## 3. Apical Uptake, Intracellular Metabolism and Basolateral Secretion of Vitamin A by the Intestinal Cell 

**Figure 2:**
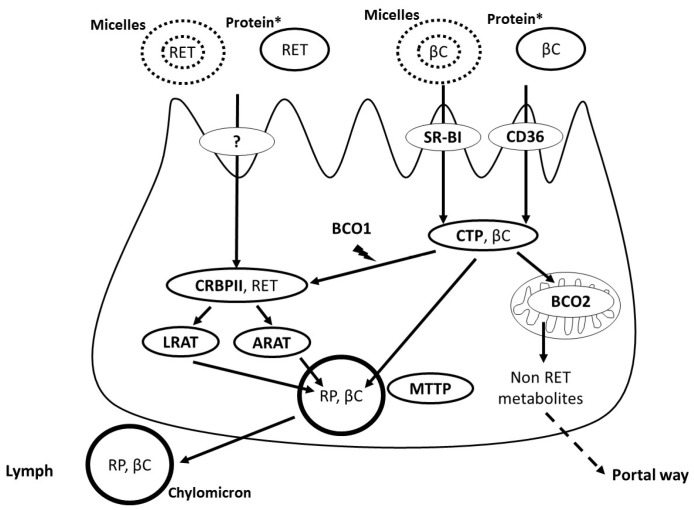
Proteins involved in vitamin A metabolism within the enterocyte. ARAT: acyl-CoA:retinol acyltransferases; βC: β-carotene and all other provitamin A carotenoids; BCO1: β-carotene oxygenase 1; BCO2: β-carotene oxygenase 2; CD36: cluster determinant 36; CRBPII: cellular retinol binding protein II; CTP: cellular transport protein (BCO1 and L-FABP are candidates); LRAT: lecithin retinol acyltransferase; MTTP: microsomal triglyceride transfer protein; RET: retinol; RP: retinyl palmitate and all other retinyl esters; SR-BI: scavenger receptor class B type I. It is assumed that there is an apical transporter of RET, but since it has not been identified, a question mark has been added.

Since mixed micelles are assumed to dissociate in the unstirred water layer adjacent to the enterocyte apical membrane [24], VA incorporated in mixed micelles, i.e., RET and βC, is supposed to reach the apical membrane as free molecules. However, the fact that scavenger-receptor class B-type I (SR-BI), which is encoded by *SCARB1*, facilitates in cell culture and in mice the uptake of several molecules with fairly different chemical structures, e.g., cholesterol [25], vitamin E [26], vitamin K [27] and carotenoids [28], does not fit with this assumption. Consequently, another hypothesis might be that this transporter interacts with mixed micelles rather than with free VA molecules and that micelle components and VA then diffuse to the apical membrane. The mechanisms by which these molecules cross this membrane and are secreted into the cytoplasm are not known. Several results suggest that both the SR-BI and CD36 molecule (CD36) are involved in proVA carotenoid uptake [28,29,30,31] (Figure 2), but not in that of RET [28]. Yet it is assumed, from the work of Hollander’s group [23,32], that RET is absorbed, at least partly, by a saturable, protein-mediated passive absorption mechanism. This membrane protein remains to be identified. 

After having crossed the apical membrane, RET and βC have to cross the polarized intestinal cell to be secreted at its basolateral side. The intracellular transport of RET is performed, at least partly, by the cellular retinol-binding protein type II (CRBPII), which is expressed solely in the adult intestine [33]. It is assumed that CRBPII transports RET to the sites where it is either oxidized to retinal and then to retinoic acid, which is involved in gene expression regulation in the enterocyte, or esterified to retinyl esters, a necessary step for its incorporation into chylomicrons. RET esterification, which occurs in the endoplasmic reticulum, has been shown to be performed by several enzymes. The main one, i.e., the enzyme that esterifies most RET in usual dietary conditions [34], is lecithin-retinol acyltransferase (LRAT), which uses RET bound to CRBPII as a substrate [35]. The other ones are acyl-CoA:retinol acyltransferases (ARAT) that esterify RET via an acyl-CoA-dependent process [34,35]. At least two enzymes that exhibit an ARAT activity are present in the enterocyte [34]. The main one is diacylglycerol acyltransferase 1 (DGAT1) [34], but there is likely at least another one. Indeed, since loss of DGAT1 activity does not completely impair RET esterification [36], this suggests that either LRAT is very efficient or, most likely, that another enzyme that exhibits an ARAT activity is present in the enterocyte [34]. The relative activity of these acyltransferases, which use different sources of intracellular fatty acids to esterify RET, i.e., LRAT uses fatty acids from intracellular membrane phospholipids, while DGAT1 and the other ARAT(s) use newly-absorbed fatty acids, is assumed to explain the variability in the pattern of retinyl esters synthetized after meals that provided a different amount and species of fatty acids [37]. Less is known about the intracellular transport and metabolism of βC in the enterocyte. Nevertheless, since it is assumed that it is not transported by CRBPII, another intracellular binding protein is likely to be involved [38]. This protein could be beta-carotene oxygenase 1 (BCO1), which is mainly located in the cytosol of mature enterocytes from the jejunum [39], because it is the main enzyme that cleaves βC [40,41,42,43], and it has a great affinity for βC. This intracellular transport protein could also be beta-carotene oxygenase 2 (BCO2), although it is apparently mainly located in mitochondria [44]. The intracellular βC transporter could also be a fatty acid binding protein (FABP), more likely liver FABP (L-FABP), which is also present in the intestine and which displays high-affinity binding for various hydrophobic ligands [45]. At this step, it is important to make clear that only a fraction of absorbed βC is metabolized in the enterocyte. The importance of this fraction, which was estimated at about 70% by using the stable isotope method [46], depends on the VA status of the body (see the next section). The fraction of non-metabolized βC is incorporated in nascent chylomicrons [47]. The exact mechanism of this incorporation is not known, but it is assumed that it involves enzymes/apoproteins responsible for the assembling of these triglyceride-rich lipoproteins, such as microsomal triglyceride transfer protein (MTP) and apoB48.

The more apolar forms of VA present in the intestinal cell, i.e., RP and βC, are assumed to be mostly secreted in chylomicrons, while the less apolar forms, i.e., RET [48], retinoic acid and apocarotenoids, are assumed to be secreted in the portal blood. The relative proportion of VA secreted in these two pathways is not known, but we suggest that it depends on the relative activities of the enterocyte enzymes involved in VA metabolism.

## 4. Regulation of Vitamin A Absorption

It is now acknowledged that VA status regulates βC absorption and cleavage efficiency via a negative feedback loop: the higher the VA status, the lower βC absorption efficiency and cleavage, and inversely. The mechanism involves an intestinal transcription factor called intestine specific homeobox (ISX), which acts as a repressor of *SCARB1* and *BCO1* upon retinoic acid activation [49,50]. Following VA uptake, the intracellular concentration of retinoic acid increases leading to the induction of ISX gene expression. Consequently, less βC is taken up by the enterocyte, and less βC is converted to retinal. When the intracellular concentration of retinoic acid drops, which is assumed to be the case when the dietary VA intake is insufficient, ISX exerts less repressor activity towards *SCARBI* and *BCO1*, and consequently, βC uptake and conversion efficiency increase. This mechanism is thought to regulate the absorption and the cleavage efficiencies of other proVA carotenoids, as well, as they are also absorbed via SR-BI and cleaved by BCO1. A study in Zambian children with hypervitaminosis A supports this regulation. Indeed, these children had high serum carotenoid concentrations [51], and many of them experienced hypercarotenodermia during mango season, a period of high carotenoid intake. This might indicate that proVA carotenoid conversion to VA by BCO1 was more inhibited by the hypervitaminosis A than their absorption via SR-BI. This is not surprising as proVA carotenoids’ absorption involves not only SR-BI, but also CD36, which is not assumed to be regulated by ISX. Finally, it is important to state that there is no study dedicated to assess whether VA status also modulates the absorption efficiency of preformed VA.

## 5. Postprandial Blood Transport of Newly-Absorbed Vitamin A from the Intestine to the Liver

The intestine is assumed to secrete most newly-absorbed VA into chylomicrons. The two main VA vitamers found in these triglyceride-rich lipoproteins are (i) RP [37], which comes either from RET re-esterification or from esterification of RET produced by enterocyte metabolism of βC, and (ii) βC that has not undergone cleavage by BCO1 or BCO2 in the enterocyte. Most of RET is secreted as retinyl esters in the chylomicrons, regardless of the chemical and physical form of administration [22]. Note that when a pharmacological dose of retinyl palmitate is ingested with a meal almost depleted in fat, chylomicrons can also contain a significant proportion of RET that has not undergone esterification in the enterocyte [37]. It has been shown that most RP and βC are not exchanged between lipoproteins and remain in chylomicron and their remnants during their intravascular metabolism [52,53]. Most VA incorporated into chylomicron remnants, which are produced during vascular lipolysis of chylomicron triglycerides by both lipoprotein lipase (LPL) and glycosylphosphatidylinositol anchored high density lipoprotein binding protein 1 (GPIHBP1) [54], is taken up by hepatocytes during the postprandial period [55]. Although most newly-absorbed VA is secreted into chylomicrons, it is assumed that the water-soluble VA metabolites, e.g., retinoic acid and apo-carotenals, could be secreted in the portal circulation and could then directly reach the liver.

## 6. Liver Metabolism of Vitamin A and Blood Transport of Vitamin A from the Liver to Extra-Hepatic Tissues 

**Figure 3:**
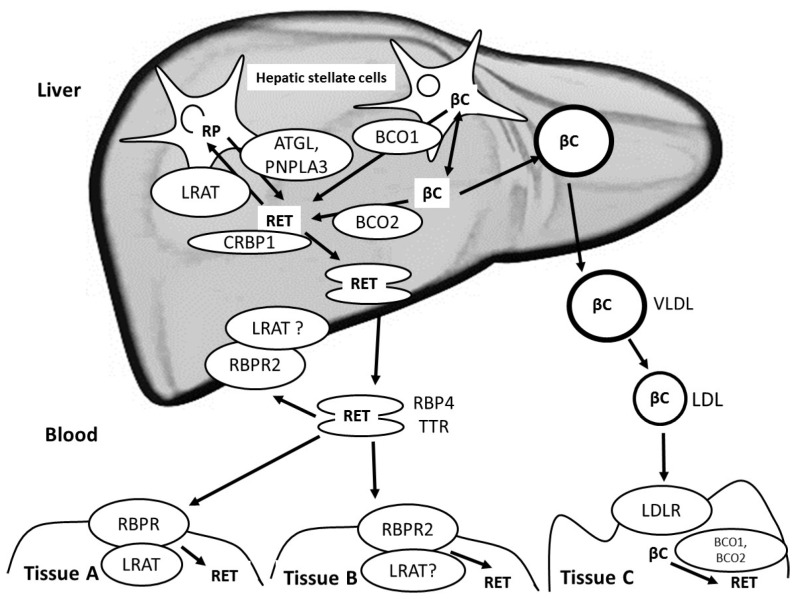
Proteins involved in the liver metabolism of vitamin A. ATGL: adipose triglyceride lipase; βC: β-carotene and all other provitamin A carotenoids; BCO1: β-carotene oxygenase 1; BCO2: β-carotene oxygenase 2; CD36: cluster determinant 36; CRBPI: cellular retinol binding protein I; LDLR: LDL receptor; LRAT: lecithin retinol acyltransferase; PNPLA3: patatin-like phospholipase domain-containing 3; RBPR2: RBP4 receptor-2; RBP4: serum retinol-binding protein; RBPR: RBP receptor (encoded by STRA6); RET: retinol; RP: retinyl palmitate and all other retinyl esters; TTR: transthyretin. The liver is the hub of vitamin A metabolism: it is the main organ that stores VA and distributes it to the peripheral tissues. VA reaches the liver mainly as retinyl esters, mainly RP, and provitamin A carotenoids, mainly βC, incorporated in chylomicrons following VA absorption. VA is then mostly stored in hepatic stellate cells. This figure shows the main proteins involved in the mobilization of the liver stores of VA and in the distribution of liver VA to peripheral tissues.

Liver is the main storage organ for VA (Figure 3). Indeed, it has been estimated that for healthy, well-nourished individuals, approximately 70% of VA present in the body is stored in the liver [56]. Following chylomicron-remnant uptake by the liver, which involves cell surface receptors, i.e., LDL-receptor, LDL-receptor related protein 1 (LRP1) and heparan sulfate proteoglycans (HSPGs) [54], it is assumed that chylomicron remnant RP and βC are released in hepatocytes during chylomicron remnant metabolism. They are then assumed to follow different metabolic pathways. RP is assumed to be hydrolyzed by a retinyl ester hydrolase (REH) to give RET. RET is then assumed to bind to cellular retinol-binding protein type I (CRBPI) [57] and be transported to either the site where it is transferred to retinol-binding protein 4 (RBP4) or to hepatic stellate cells where it is esterified by LRAT [58,59]. Interestingly though, hepatic LRAT expression is regulated by VA status [55]. This regulation likely involves retinoic acid and its response elements, i.e., retinoic acid receptor (RAR) and/or retinoid X receptor (RXR). This regulation is proposed to give rise to a positive feedback loop when cellular retinoic acid concentrations are high, turning on hepatic stellate cell LRAT expression [60] and increasing the synthesis of retinyl esters [56] in these cells [61,62], which are also called fat-storing cells, lipocytes or Ito cells. These cells store approximately 70%–90% of liver VA [56]. The mobilization of retinyl ester stores is performed by at least two lipases: adipose triglyceride lipase (ATGL) [63] and patatin-like phospholipase domain-containing 3 (PNPLA3) protein [64], which has also a triglyceride hydrolase activity [65,66]. Conversely to that of chylomicron RP, the fate of chylomicron βC in the liver is barely known. Indeed, how βC is released from chylomicrons and how it is transported into hepatocytes remains unanswered. Concerning its cleavage, it is assumed that it is either cleaved to retinal by BCO1, which is highly expressed in hepatic stellate cells [67], or BCO2, which is apparently more expressed in hepatocytes [67]. The fraction of βC that does not undergo this cleavage is either incorporated into very low density lipoproteins (VLDL), which are then secreted in the blood, or stored in lipid droplets in parenchymal cells and in hepatic stellate cells [67,68]. The mechanism involved in the mobilization of these βC stores is not known, but we hypothesize that it requires the hydrolysis of lipid droplet triglycerides.

The liver secretes VA either in the bile, as oxidized and/or conjugated metabolites [69,70], or in the blood. Two main forms of VA are secreted in the blood: RET and βC. RET is bound to serum retinol binding protein (sRBP, RBP4), which in turn binds to transthyretin (TTR), stabilizing the complex [71]. βC is incorporated in VLDL. RET associated with RBP4/TTR is taken up by two structurally-related membrane receptors: stimulated by retinoic acid 6 (STRA6) [72] and the recently discovered STRA6-like, also known as RBP4 receptor-2 (RBPR2) [73]. Retinol uptake via STRA6 depends on a functional coupling with intracellular LRAT [74]. STRA6 and RBPR2 exhibit different tissue expression patterns: STRA6 is expressed in numerous tissues, but not in liver and intestine, where RBPR2 is mostly expressed [73]. VLDL-βC and low density lipoprotein (LDL)-βC, which originate from VLDL metabolism, are most likely taken up via the LDL-receptor-dependent mechanisms [75], requiring the tissue/organs to express the LDL-receptor.

## 7. Vitamin A Metabolism in Extra-Hepatic Tissues

VA plays a critical role as a signaling molecule in most tissues [76,77] and chromophore in eyes [2,3,4,5]. The metabolism of VA in extra-hepatic tissues is assumed to involve pathways like those present in the liver, e.g., RET is assumed to give retinal, then retinoic acid; βC is assumed to be cleaved into retinal and/or apocarotenals, although some differences do exist. Another example concerns the hydrolysis of retinyl esters. Indeed, while hormone-sensitive lipase (HSL) is the predominant retinyl ester hydrolase in adipocytes [63,78], it is apparently not involved in retinyl ester hydrolysis in the liver [63]. Nevertheless, it is out of the topic of this review to comprehensively describe the metabolism of VA in all extra-hepatic tissues.

## 8. Physiological Regulation of Blood Vitamin A Concentrations

Numerous forms of VA circulate in human blood: RET, proVA carotenoids, retinyl esters, retinoic acid, retinyl-β-glucuronide, retinoyl-β-glucuronide [56]. Furthermore, the blood concentration of these various VA forms can significantly vary in the postprandial period as compared to the fasting state. Thus, when talking about the blood VA concentration, it is important to specify which VA molecule is meant and when its concentration is measured, i.e., in the fasting state or during the postprandial period. It is assumed that the blood concentration of these different forms of VA is differently regulated by our body. In this review, we have decided to focus on the regulation of the three main forms of VA, in terms of absolute concentration, recovered at fast and in the postprandial period, i.e., RET, βC and retinyl esters.

Concerning RET concentration, it is acknowledged that it is tightly regulated [79] with concentrations ranging from 2 to 4 µM at fast in adults [80]. Its concentration only changes in response to extreme VA dietary events or in disease states [79], and in the postprandial period when a meal rich in VA is provided to subjects deficient in VA. This last observation is assumed to be due to the fact that when hepatic VA stores are very low, free RBP accumulates in the liver [81]. When a high amount of VA then reaches the liver, usually following the consumption of a high dose of VA, it binds the free RBP accumulated, and it is quickly released in the blood, leading to a transitory increase in blood RET [82]. This has led to developing two tests that are used to evaluate the VA status: the relative dose response (RDR) test [82,83] and the modified relative dose response test (MRDR) [84,85]. 

Regarding the βC blood concentration, it is acknowledged that there is no direct regulation. Indeed, this form of VA is found in all lipoprotein classes [86,87,88], including chylomicrons during the postprandial period when a meal containing βC is ingested. Thus, blood concentration of βC depends on: (1) the state at which the blood is collected, i.e., at fast or in the postprandial period; (2) the amount of βC that was ingested in the previous meal; and (3) on the metabolism of lipoproteins in which it is incorporated and, thus, on the regulation of lipoprotein metabolism. 

Concerning retinyl esters, although it has been suggested that some are recovered in VLDL and LDL [56], most of them are incorporated in chylomicrons after VA absorption and intestinal VA metabolism. Thus, it is assumed that there is no direct regulation of their blood concentration. Indeed, retinyl esters are assumed to stay within the chylomicrons during their blood metabolism, and thus, their blood concentration exhibits a bell-shaped curve [47] that closely mimics that of chylomicron triglycerides. For this reason, retinyl esters are assumed to be a valuable marker of chylomicrons and their remnants [89]. Thus, blood retinyl ester concentration during the postprandial period is assumed to be governed by the factors that regulate the metabolism of chylomicrons, i.e., those that govern their intestinal secretion, blood metabolism and uptake by the liver [54,90,91].

Overall, it can be concluded that blood VA concentration is modulated by the activity of numerous proteins, e.g., those that participate in the secretion and blood metabolism of chylomicrons and other lipoproteins, regarding βC and RP, and those that participate in the liver secretion and blood metabolism of RET.

## 9. Genetic Variations that Have Been Suggested to Modulate Blood Vitamin A Concentrations

The measurement of liver stores of VA is considered the gold standard to assess an individual’s VA status. However, there is no non-invasive method to date, and thus, the use of alternative biomarkers is required (see [79] for a recent review thereof). Serum RET concentration is homeostatically regulated and only correlates with liver VA stores in the case of deficiency. Moreover, it can be affected by current or recent acute infections or chronic inflammation. Consequently, the World Health Organization does not recommend its use to assess the VA status of individuals, although it is still useful at the population level [92]. Retinol isotope dilution [93] is a quantitative and sensitive method to assess VA status over a wide range of liver VA stores. Since methods to assess VA status remain expensive, the use of GWAS to identify genetic variations associated with VA status is a great challenge, and consequently, the information on the influence of genetic variations on VA status is scarce. Only one study has reported the association of an SNP with liver stores of VA: Kovarova et al. [94] showed that an SNP in *PNPLA3*, a gene involved in the mobilization of retinyl esters stored in stellate cells [64], was associated with increased RP liver storage in a group of 42 patients undergoing liver surgery. Interestingly, the minor allele of the SNP is highly prevalent in populations from Latin American (about 70%), whereas it is found at a much lower frequency (around 20%) in populations from Europe and Africa [95]. We suggest that the effect of this SNP could be due both to a lower hydrolysis of liver RP, but also to a lower hydrolysis of intracellular triglycerides that solubilize RP. Indeed, it has been demonstrated that a loss-of-function mutation in *PNPLA3* impairs triglyceride hydrolysis [65] and promotes intracellular lipid accumulation by reducing the lipidation of VLDL [96]. Furthermore this mutation, as well as mutations in *ATGL*, which is the other lipase known to hydrolyze both RP and triglycerides [63], are genetic determinants of chronic liver diseases [97,98,99].

### 9.1. Genetic Variations Associated with Fasting Blood Vitamin A Concentrations

RET and βC are the two most concentrated VA forms in the fasting blood. Their concentration is used to assess VA or βC status, respectively. Although their usefulness for assessing the VA status of individuals is questionable, they are still acknowledged to provide valuable information on the VA status of a population [92]. Since populations with different dietary habits likely have different VA metabolism, because of adaptation and evolution, it is relevant to study the contribution of genetic variations to this phenomenon. Indeed, this might lead to provide recommended dietary allowances, or nutritional/cooking advice, more fitted to the genetic specificity of ethnic groups. Fewer than 10 studies are available on the effect of genetic variations on VA metabolism, and most of them are dedicated to associations with blood concentrations of RET or βC.

Concerning RET, the first study showing an association between a genetic variation and the blood concentration of its blood binding protein, i.e., RBP4, was published in 1995 [100]. It showed that genetic variations causing amino acid substitutions at position 84 of the TTR molecule (Ser84 and Asn84) led to substantial decreases in blood concentrations of RBP4. Four years later, two mutations in *RBP4* in two German sisters were associated with extremely low, i.e., <0.19 μmol/L, blood RET concentrations although the authors reported a partial cellular supply of retinol from circulating retinyl esters [101]. It was further shown that, although the mutant proteins can form complexes with retinol and TTR in vitro, the mutated retinol-RBP complexes are significantly less stable than normal retinol-RBP complexes, which in turn can lead to the lowering of plasma retinol and RBP concentrations [102]. A GWAS has confirmed that SNPs in the two genes encoding the proteins that transport RET in the blood, i.e., RBP4 and TTR, can significantly affect blood RET concentrations [103]. Nevertheless, it appears that fasting blood RET concentration can also be modulated by genetic variations in proteins/enzymes located in tissues. Indeed, an association between SNPs in *BCO1* and blood RET was found [104], suggesting that provitamin A carotenoids significantly participate in blood RET concentrations. Finally, a recent study has found an association between an SNP in *PNPLA3* and blood RET concentration in patients with non-alcoholic fatty liver disease or obesity [105]. Of note, the same SNP has been associated in another study with increased RP liver storage [94].

Concerning fasting blood βC concentration, one GWAS [106] and two candidate gene association studies [104,107] have shown that SNPs in *BCO1*, the main βC metabolizing enzyme, were associated with its circulating concentration. Candidate gene association studies have also found SNPs associated with blood βC concentration in three other genes. These genes were *LPL* [108], which encodes for lipoprotein lipase, a lipase involved in vascular metabolism of lipoproteins, which transport among other molecules βC, hepatic lipase (*HL)* [109], which encodes hepatic lipase, another vascular lipase involved in lipoprotein metabolism, and *SCARB1* [110], the gene that encodes for SR-BI, a membrane protein that participates in the cellular uptake of HDL [111], which can carry βC, and in the uptake of βC by enterocytes [28,30,112].

### 9.2. Genetic Variations Associated with Postprandial Blood Vitamin A Concentrations

Although the identification of genetic variations associated with fasting blood RET or proVA carotenoid concentrations is relevant to better understand VA metabolism, the application of the results to clinical practice or dietary recommendation is otherwise not straightforward due to two main reasons. First, as mentioned above, fasting blood RET concentration does not constitute a good biomarker of VA status of individuals due to its very tight homeostatic control. Second, since the highest risk factor for developing VA deficiency or insufficiency is usually low VA intakes and since proVA carotenoids display a wide-ranging bioavailability (data are lacking regarding preformed VA bioavailability), which is partly due to genetic variability, the fight against VA deficiency should rely on identifying tailored nutritional strategies, i.e., based on the assessment of the preformed VA/proVA carotenoid responder phenotype of a population/individual. For example, an individual or a group of individuals exhibiting VA deficiency (or at risk) with low capacity to absorb and/or convert proVA carotenoids should be given preformed VA supplements. The knowledge of a genotype associated with low fasting blood RET or proVA concentrations only provides little information regarding the best nutritional strategy to adopt to avoid VA deficiency, and it is thus more relevant to search for genetic variations associated with postprandial blood VA concentrations, a marker of the preformed VA/proVA carotenoid responder phenotype.

As stated above, in the postprandial period, blood can contain the three main forms of VA, depending on the source of VA that was ingested in the preceding meal and on the VA status of the subject. Indeed, following a meal containing only preformed VA, the blood contains RP incorporated in chylomicrons plus all of the other VA species that circulate at fast, i.e., mainly RET and proVA carotenoids. Following a meal containing proVA carotenoids, the blood contains RP plus the non-cleaved fraction of proVA carotenoids in chylomicrons, plus all of the other VA species that circulate at fast.

For the time being, there are only three studies dedicated to identifying genetic variations associated with postprandial blood VA concentration (Table 1). Since the measurement of this phenotype requires that volunteers stay in a clinical environment for several hours with repeated blood collections, the number of subjects included in this kind of study is usually relatively low (typically < 100). This precludes the use of GWAS, due to lack of power, and requires the use of candidate gene association studies to identify genetic variants involved. These studies were dedicated to βC metabolism and measured either postprandial chylomicron βC [113] or postprandial triglyceride-rich lipoprotein βC and RP concentrations [114,115] after test meals that provided βC. Thus, there is still no data on genetic variations associated with postprandial blood RP concentrations after a test meal rich in preformed VA. Consequently, it is obvious that the observations/conclusions presented in this chapter will be significantly improved in the future. In the study dedicated to identifying genetic variations associated with βC bioavailability [113], it was observed that the variability in the postprandial blood βC response (Area under the curve of the 0–8 h postprandial chylomicron βC concentrations) was associated with a combination of 25 SNPs in or near 12 candidate genes. Four of these genes were involved in the postprandial chylomicron triacylglycerol response in the same group of subjects [91], which was not surprising, as newly-absorbed βC is carried from the intestine to the liver via chylomicrons. Nevertheless, eight of these genes were specifically associated with the βC response. Possible explanations why these genes were associated with the βC response are discussed in the original paper [113]. Nevertheless, two associations deserve closer attention. The association with *ISX* confirms that genetic variations in this gene, which encodes a transcription factor involved in βC intestinal absorption and conversion, are key determinants of blood βC concentrations. Indeed, it was also reported in another study that an SNP in the ISX binding site in the *BCO1* promoter (rs6564851) was associated with decreased conversion rates of βC by 50% and increased fasting blood concentrations of βC [49]. The association with *BCO1* supports associations observed with postprandial βC and RP responses in other studies [114,115], confirming that this gene and its variations are key regulators of blood concentrations of these VA forms.

## 10. Other Genetic Variations that Could Be Involved in the Blood Concentration of Vitamin A

The few available studies reviewed in the two previous paragraphs highlights the tremendous work that remains to be done to identify all of the genetic variations associated with the concentration of the different forms of VA that circulate in the blood at fast and during the postprandial period. Furthermore, there is no study dedicated to identifying genetic variations that could modulate VA concentration in different tissues. It should also be reminded that, although SNPs represent >96% of polymorphisms [95], other genetic variations occur in DNA, e.g., copy number variants, insertion/deletion of some base pairs, as well as epigenetic modifications. A genetic score that would aim to predict concentrations of the different forms of VA in blood and in different tissues should therefore consider all of the genetic variations that can have a significant impact on these concentrations. Finally, association studies must be performed in different populations to be sure that the associations are not specific to some ethnic groups.

In summary, there is now enough evidence to state that blood, and likely tissue, concentrations of the different forms of VA, as well as VA bioavailability, are partly modulated by SNPs in several genes. However, much work remains to be done to obtain combinations of genetic variations (SNPs, but also other kinds of genetic variations) that will allow us to confidently predict the concentration of VA in the blood or in a target tissue of an individual by knowing his/her genotypes at these variations. Yet, the potential usefulness of this area of research is exciting regarding personalized nutrition and the fight against VA deficiency. Nevertheless, it should be reminded that genetics only represents one of the factors that affect VA concentration in blood and tissues, albeit stable over the lifespan, since other factors, such as VA dietary intake and factors that affect VA bioavailability (e.g., cooking practice), also affect this status. Thus, a prediction of VA concentration in blood and in various tissues should consider these variables, as well.

## 11. Conclusions

This review shows that genetic variations modulate both fasting blood retinol and βC concentrations and βC bioavailability. It also allows us to realize that a lot of work remains to be done to identify all the genetic variations that modulate these phenotypes and to propose a genetic test that will allow us to predict VA status or VA bioavailability in different ethnies. 

## Figures and Tables

**Table 1:** Summary of SNPs associated with fasting blood vitamin A concentration or vitamin A bioavailability.

SNP/Mutation	Global MAF ^1^	Nearest Gene	Trait	Reference	Study Type
(Ile59Asn) (rs121918584)	-	*RBP4*	FB-RET	[101,102]	CS
Gly75Asp (rs1218585)	0.115	*RBP4*	FB-RET	[101,102]	CS
c.248 + 1G>A	-	*RBP4*	FB-RET	[116]	CS
rs10882272	0.390	*RBP4*	FB-RET	[103]	GWAS
rs1667255	0.500	*TTR*	FB-RET	[103]	GWAS
rs738409	0.2622	*PNPLA3*	FB-RET	[105]	CGAS
rs6564851	0.476	*BCO1*	FB-βC	[104,106,107,114,117]	GWAS and CGAS
rs12926540	0.493	*BCO1*	FB-βC	[117]	GWAS
rs7501331	0.213	*BCO1*	FB-βC	[104,115]	CGAS
rs12934922	0.357	*BCO1*	FB-βC	[104,115]	CGAS
rs1800588	0.292	*HL*	FB-βC	[109]	CGAS
S447X	-	*LPL*	FB-βC	[108]	CGAS
SR-BI intron 5	-	*SCARB1*	FB-βC	[110]	CGAS
rs61932577	0.033	*SCARB1*	FB-βC/αC	[28]	CGAS
rs1984112	0.347	*CD36*	FB-βCryt/αC	[28]	CGAS
rs1761667	0.390	*CD36*	FB-βCryt/αC	[28]	CGAS
rs7755	0.388	*CD36*	FB-βCryt/αC	[28]	CGAS
rs10991408 *	0.116	*ABCA1*	βC-B ^2^	[113]	CGAS
rs2791952 *	0.140	*ABCA1*	βC-B	[113]	CGAS
rs3887137 *	0.123	*ABCA1*	βC-B	[113]	CGAS
rs2278357	0.247	*ABCG5*	βC-B	[113]	CGAS
rs1042031 *	0.153	*APOB*	βC-B	[113]	CGAS
rs35364714 *	0.115	*APOB*	βC-B	[113]	CGAS
rs4643493 *	0.082	*APOB*	βC-B	[113]	CGAS
rs7196470	0.278	*BCO1*	βC-B	[113]	CGAS
rs1247620	0.137	*CXCL8*	βC-B	[113]	CGAS
rs1358594	0.291	*CXCL8*	βC-B	[113]	CGAS
rs6834586	0.221	*CXCL8*	βC-B	[113]	CGAS
rs3798709	0.252	*ELOVL2*	βC-B	[113]	CGAS
rs911196	0.252	*ELOVL2*	βC-B	[113]	CGAS
rs9468304	0.302	*ELOVL2*	βC-B	[113]	CGAS
rs16994824	0.206	*ISX*	βC-B	[113]	CGAS
rs202313	0.113	*ISX*	βC-B	[113]	CGAS
rs5755368	0.250	*ISX*	βC-B	[113]	CGAS
rs11857380 *	0.157	*LIPC*	βC-B	[113]	CGAS
rs12185072 *	0.198	*LIPC*	βC-B	[113]	CGAS
rs1869138 *	0.117	*LIPC*	βC-B	[113]	CGAS
rs8043708	0.237	*PKD1L2*	βC-B	[113]	CGAS
rs12139131	0.096	*RPE65*	βC-B	[113]	CGAS
rs4926340	0.093	*RPE65*	βC-B	[113]	CGAS
rs2501175	0.327	*SOD2*	βC-B	[113]	CGAS
rs946199 *	0.192	*TCF7L2*	βC-B	[113]	CGAS

^1^ Abbreviations: CS: case subject(s) with very low vitamin A status; CGAS: candidate gene association study; GWAS: genome-wide association study; MAF: minor allele frequency, retrieved from the SNP database in PubMed (https://www.ncbi.nlm.nih.gov/pubmed?cmd=search); the gene official symbols are those found in PubMed and approved by the Hugo Gene Nomenclature Committee (available online: http://www.genenames.org/). FB-βC: fasting blood β-carotene concentration; FB-βCrypt/αC: fasting blood β-cryptoxanthin or α-carotene; FB-RET: fasting blood retinol concentration; βC-B: β-carotene bioavailability. ^2^ In this study, β-carotene bioavailability was estimated by measuring the postprandial chylomicron β-carotene response (0–8 h area under the curve) to a β-carotene containing test-meal. * These SNPs were associated with the variability of β-carotene bioavailability, but this association was likely due to their involvement in the postprandial metabolism of chylomicrons [91,113], which are the lipoparticles that carry newly-absorbed β-carotene from the intestine to the liver.
